# Multi-omic approach to identify risk markers specific to COVID-19

**DOI:** 10.1016/j.ebiom.2022.104009

**Published:** 2022-04-19

**Authors:** Won-Young Kim

**Affiliations:** Division of Pulmonary and Critical Care Medicine, Department of Internal Medicine, Chung-Ang University Hospital, Chung-Ang University College of Medicine, 102 Heukseok-ro, Dongjak-gu, Seoul 06973, Republic of Korea

Coronavirus disease 2019 (COVID-19) has infected more than 470 million people and caused more than 6 million deaths worldwide. The high mortality mainly owes to the associated acute respiratory distress syndrome (ARDS), which is characterised by the sudden onset of noncardiogenic pulmonary oedema and hypoxemia.^1^ Recent studies have revealed that COVID-19 ARDS is a dysregulated host response of inflammation, immunity, and interferon signalling.^2^ Increasing evidence also suggests that severe acute respiratory syndrome coronavirus 2 (SARS-CoV-2) exerts detrimental effects on the capillary endothelium, possibly by altering the integrity of the endothelial barrier or promoting a pro-coagulant state and the resulted endothelial inflammation.^3^ In addition to lung damage, sepsis and similar organ dysfunctions are also common, which further contribute to the high mortality of severe COVID-19.^4^ As severe COVID-19 shares common characteristics with ARDS or sepsis, the complex immune response cannot be easily distinguished from each other.

In this issue of *eBioMedicine*, Gustafson and colleagues prospectively evaluated the prevalence of plasma inflammatory, cardiac, and endothelial cell biomarkers in 241 unvaccinated patients with suspected SARS-CoV-2 infection and established a microRNA (miRNA) atlas.^5^ Using COVID-19 severity and two symptom/severity-matched control groups,^6^ the authors first defined 5 patient groups: mild negative, mild COVID-19, moderate COVID-19, severe COVID-19, and severe negative. For risk assessment, a Random Forest model machine learning was performed utilizing clinical, protein marker, and miRNA data. Among protein biomarkers, Ang-2, ET-1, sICAM-1, sVCAM-1, sE-selectin, sTREM-1, IL-6, IL-8, and MPO levels differed according to COVID-19 severity. However, there were no significant differences between the severe COVID-19 and severe negative groups.

Among hospitalised patients, only Ang-2 was associated with mortality in univariate analysis. Moreover, only IL-6 and MPO remained significantly different between the severe COVID-19 and severe negative groups during the course of illness. These findings suggest that protein biomarkers of endothelial dysfunction and inflammation may not be specific to COVID-19 status or mortality. Meanwhile, comparative analysis revealed 765 miRNAs that could be used for differentiation between the severe COVID-19 and severe negative groups; these included disease-relevant miRNA pathways for cardiomyocyte function and adherens junctions. In addition, there were 207 differentially expressed miRNAs between survivors and non-survivors in the severe COVID-19 group; these included miRNA pathways for platelet activation, extracellular matrix-receptor interactions, Ras, and ErbB2. Clinical data alone at the time of admission had low predictive capability for risk of hospital mortality (AUROC: 0·44). However, the addition of protein marker and miRNA data enhanced the model performance (AUROC: 0·82 and 0·76, respectively).

Next, the authors performed *ex vivo* experiments to assess endothelial permeability in patient plasma. Interestingly, they found endothelial barrier disruption in response to plasma from moderate and severe COVID-19 patients, but not to plasma from severe negative patients. Administration of Q-peptide, synthetic Ang-1, or recombinant Slit2-N ameliorated the disruption of the endothelial barrier. However, administration of other agents (nangibotide and dexamethasone) had no such effect.

Clinical trials have shown a significant survival benefit for dexamethasone in patients with COVID-19 ARDS^7^ while inconsistent results were reported for non-COVID-19 ARDS.^1^ The different results suggest distinct pathogenesis for COVID-19 ARDS. While endothelial cell biomarkers have shown utility in COVID-19 prognostication,^8^ it seems unlikely that a simple combination of biomarkers can characterise the pathophysiological alterations in COVID-19 patients or individualise management according to the immune phenotypes. The study by Gustafson and colleagues is important because the incorporation of clinical data with multi-omic approaches identified unique COVID-19 phenotypes and provided prognostic information and mechanistic evidence.^9^ The authors should also be credited for demonstrating the failure of dexamethasone in stabilizing barrier function, which is consistent with preclinical studies that suggest glucocorticoids may impair endothelial function by decreasing vascular NO availability.^10^ Further, glucocorticoids are only beneficial under inflammatory conditions, possibly due to decreased expression of IL-6, IL-8, VEGF, endothelin-1, and NF-kB.^10^ The present study reinforces the notion that the timing and setting are important for therapeutic interventions.

There are, however, important limitations. The associations between biomarkers and clinical outcomes should be interpreted with caution due to the modest sample size, missing data, and inability to perform multivariable analysis. Moreover, although the severe negative group demonstrated symptoms consistent with a respiratory tract infection and were matched by illness severity, the group showed somewhat less severe (respiratory) symptom than that of the severe COVID-19 group. Further, compared with the severe negative group, the severe COVID-19 group had longer intensive care unit stays, and they were more likely to have ARDS and worse oxygenation. Indeed, the higher SOFA respiratory subscore in these patients might lead to biased results. For instance, the barrier disruption in response to severe COVID-19 patient plasma did not occur in plasma from severe negative patients. This may be due to the lower disease severity in the severe negative patients. It could be argued that any difference in the clinical trajectories between severe COVID-19 patients and severe negative patients is related to the biology of COVID-19. However, it is questionable that the study patients who presented with respiratory sepsis were clinically indistinguishable at the time of enrolment.

Nevertheless, to our best knowledge, this is the first report to provide a comprehensive, multi-omics-based description of immune markers for risk stratification specific to COVID-19. The study offers novel insights with regard to the role of endothelium and endothelial barrier stabilizing treatments in COVID-19. Further studies with larger sample sizes and proper controls are needed to validate the results.

## Contributors

WYK wrote the paper.

## Declaration of interests

The author declares no conflict of interest.
